# Adult and young women communication on sexuality: a pilot intervention in Maputo-Mozambique

**DOI:** 10.1186/s12978-019-0809-3

**Published:** 2019-09-18

**Authors:** Mónica Frederico, Carlos Arnaldo, Kristien Michielsen, Peter Decat

**Affiliations:** 1 International Centre for Reproductive Health (ICRH), Department of Public Health and Primary Care, C. Heymanslaan 10, 9000 Ghent, UZ Belgium; 2 Centro de Estudos Africanos, Departmento de Estudos de Desenvolvimento e Género, Universidade Eduardo Mondlane, Moçambique, Julius Nyerere Ave., Main Campus, P.O.Box 1993, Maputo, Mozambique; 3 Department of Public Health and Primary Care, C. Heymanslaan 10, 9000 Ghent, Belgium; 4 Departmento de Estudos de Desenvolvimento e Género, Centro de Estudos Africanos, Universidade Eduardo Mondlane, Moçambique, Julius Nyerere Ave., Main Campus, P.O.Box 1993, Maputo, Mozambique

**Keywords:** Sexuality, Communication, Maxaquene, Maputo, Adult and young women

## Abstract

**Background:**

Communication on sexuality within the family has been considered a determinant factor for the sexual behaviour of young women, contributing to delaying sexual initiation. Taking into account that young women are increasingly exposed to sexualized messages, they need clear, trustful and open communication on sexuality more than ever. However, in Mozambique, communication about sexuality is hampered by strict social norms. This paper evaluates the case of an intervention aimed at reducing the generational barrier for talking about sexuality and to contribute to better communication within the family context.

**Methods:**

The intervention consisted of three weekly one-hour coached sessions in which female adults and young interacted about sexuality. Realist evaluation was used as a framework to assess context, mechanisms, and outcomes of the intervention. Interviews were conducted among 13 participants of the sessions.

**Result:**

The interaction sessions were positively appreciated by the participants and contributed to change norms and attitudes towards communication on sexuality within families. Recognition of similarities and awareness of differences were key in the mechanisms leading to these outcomes. This was reinforced by the use of visual materials and the atmosphere of respect and freedom of speech that characterized the interactions. Limiting factors were related to the long-standing taboo on sexuality and existing misconceptions on sexuality education and talks about sex.

**Conclusion:**

By elucidating mechanisms and contextual factors our study adds knowledge on strategies to improve transgenerational communication about sexuality.

## Resumo

**Contexto:**

A comunicação sobre sexualidade dentro da família tem sido considerada um fator determinante para o comportamento sexual de mulheres jovens, pelo facto de contribuir para retardar o início da actividade sexual. Tendo em conta que as mulheres jovens estão cada vez mais expostas a mensagens sobre o sexo, elas precisam de uma comunicação clara, confiável e aberta sobre sexualidade. No entanto, em Moçambique, esta comunicação sobre sexualidade é dificultada por normas sociais. Este artigo faz avaliação de um caso de intervenção piloto que visava reduzir as barreiras existentes entre gerações para falar sobre sexualidade e ao mesmo tempo contribuir para uma melhor comunicação sobre sexualidade no contexto familiar.

**Metodos:**

A intervenção consistiu em três sessões semanais de uma hora cada, nas quais mulheres adultas e jovens interagiram sobre sua experiência no âmbito da sexualidade. A avaliação realista foi usada como uma ferramenta para avaliar o contexto, os mecanismos e resultados da intervenção. Para tal, foram entrevistadas 13 participantes das sessões de intervenção.

**Resultados:**

As sessões de interação foram apreciadas positivamente pelos participantes e contribuíram para mudar normas e atitudes em relação à comunicação sobre sexualidade dentro das famílias. O reconhecimento de semelhanças e a consciência das diferenças entre ambas mulheres (adultas e jovens) foram aspectos fundamentais que levaram a estes resultados. Isto foi reforçado pelo uso de materiais visuais e pelo ambiente de respeito mútuo e liberdade de expressão que caracterizaram as sessões de interação. O tabu que rodeia a sexualidade e os equívocos sobre educação sexual e conversas sobre sexo, foram os fatores limitantes durante as sessões.

**Conclusão:**

Ao elucidar os mecanismos e factores contextuais, o nosso estudo agrega conhecimento sobre estratégias para melhorar a comunicação sobre sexualidade entre gerações.

## Resumen

**Contexto:**

Comunicación sobre sexualidad dentro de la familia está considerado como un determinante para la conducta sexual de jóvenes y resulta entre otros en una demora del debut sexual. Tomando en cuenta que mujeres jóvenes están cada vez más expuestas a mensajes sexualizados, ellas necesitan más que nunca una comunicación clara, confiable y abierta sobre sexualidad. A pesar de ello, en Mozambique, la comunicación sobre la sexualidad esta obstaculizada por normas sociales. Este articulo evalúa una intervención pilota que tiene como objetivo de reducir las barreras generacionales para hablar sobre sexualidad y de contribuir a una mejor comunicación dentro del contexto familiar.

**Método:**

La intervención incluyo tres sesiones semanales durante las cuales mujeres adultas y mujeres jóvenes interactuaron sobre sexualidad. Una evaluación realista ha sido aplicada como metodología para entender contextos, mecanismos y resultados de la intervención. Trece participantes a las sesiones han sido entrevistadas.

**Resultados:**

Las sesiones de interacción fueran apreciadas por las participantes y contribuyeron a cambiar normas e actitudes hacía la comunicación sobre el sexo dentro de familia. El reconocimiento de similitudes y la conciencia de diferencias fueron mecanismos claves para llegar a los resultados. Lo que fue reforzado por el uso de materiales visuales y el ambiente de respeto y de libertad de expresión que caracterizaron las interacciones. Factores limitantes fueron relacionados a los tabús persistentes sobres sexualidad y las equivocaciones comunes sobre educación sexual y la comunicación sobre el sexo.

**Conclusión:**

Clarificando mecanismos y factores contextuales, nuestra investigación ha agregado conocimiento sobre estrategias para mejorar la comunicación transgeneracional sobre sexualidad.

## Plain English summary

Conversation on sexuality issues, such as puberty, menstruation, dating, pregnancy, contraception, and abortion between mother/father and their daughters/sons is important because it helps them to prevent early sexual initiation. Given the existence of several sources of sexualized messages, young people, especially young women need clear, trustful and open communication on sexuality issues within the household context. However, in Mozambique, culturally established norms make this difficult. This paper is about the experience of a conversation about sexuality between female adults and young implemented in one of the municipal districts of the Maputo city, in Mozambique. This experience consisted of three one-hour per week sessions of conversation between young and adult women. During the sessions, female adults and young women interacted about sexuality issues. The experience was classified as positive and contributed to a change in norms and attitudes about conversations on sexuality within families. Female adults and young recognized that they have similarities, but they also were aware of their differences. This aspect was important for exchanging their experiences. The use of visual materials, respect, and freedom of speech helped the interactions. The barrier on this experience was related to taboo and beliefs in sexuality education and talks about sex. By explaining the drivers and condition under which this experience occurred, this study adds knowledge on strategies to improve conversations about sexuality between adults and young women.

## Background

The communication on sexuality within the family context has been considered a determinant factor for sexual behaviour of young women [1–4] since the family provides the role models and standards of sexual conduct [5]. Evidence shows that good parent-adolescent communication on sexuality delays sexual initiation [6–9], and contributes to healthy decision-making related to sexuality and relationships [10, 11]. Young women are increasingly exposed to sexualized messages from peers, media, television (soap opera), and the internet. Consequently, they need a clear, trustful and open communication on sexuality.

However, studies in East-African countries have shown that communication on sexuality issues in the family context between adults and adolescents is almost inexistent [12, 13]. If any, the messages about sexuality are usually based on fear, which may discourage adolescents from asking for information [12, 13]. A variety of factors hinder sexuality communication between parents and adolescents: gender differences, level of education, religion [14, 15], traditional norms which establish who can and cannot provide information about sexuality to young women [12, 14, 16, 17], and the fear of the parents to make young women curious and push them to premarital sex [12, 16, 18–21].

Several interventions have been implemented in order to overcome those obstacles. An intervention to stimulate improved household sexual communication in rural South Africa led to improved communication on sexuality within families and a content shift from vague to concrete messages on sexuality [22]. Also, an assessment of a one-year-intervention about Families Matter Program (FMP), designed to improve parent-child communication about sexual risk reduction and parenting skills in rural Western Kenya demonstrated a more positive attitude of parents regarding sexuality education, a reinforcement of the level of parental monitoring and an improved relationship between parents and adolescents [23].

In Mozambique, a multi-component initiative*, Programa Geração Biz*, a well-known and successful intervention implemented since 1999, reaching out young people in health facilities, schools, and in communities [24–26], improved young people’s knowledge of contraceptive methods and prevention of STIs and HIV. However, this intervention targets adolescents and youths outside their family context and does not focus on promoting intergenerational communication on sexuality. Thus, there is a need to know how sex dialogue between adolescents and adults could be improved in Mozambique. This paper describes the evaluation of an intervention promoting communication about sexuality between female adults and young women, implemented in Maxaquene A, Maputo. The intervention was based on the hypothesis that coached interaction between adults and adolescents reduces generational barriers for talking about sexuality and contributes to better communication within families.

## Methods

### Study setting

This intervention study was implemented within a broader research project on *Pregnancy during Adolescence and youth: analysis of the factors influencing abortion decision-making and utilization of reproductive health services in Maputo and Quelimane cities, Mozambique.* This study was conducted in Maxaquene “A”, a neighborhood located in the Municipal District of KaMaxaquene, in Maputo city, the capital of Mozambique. This setting was selected because it had a significant proportion of pregnancies (36% of the 226); and abortions (38,5% of the 26), among women aged 15 to 24 in the 2016 household survey conducted in the context of the broader research project mentioned above.

According to the 2007 census, Maxaquene “A” has a total population of 22,750 inhabitants, distributed by 4349 households of 4 members on average. About 51% of the total population are women, 56% of the households were poor and 51.9% of the population aged 15 and above were illiterate, of which 66.7% are women [27]. Almost all households had access to piped water and electricity. However, the quality of housing was poor. The main source of income for the households is small businesses such as selling food, water, vegetables, and other basic need products, near their residences or at the local informal market.

### The theory and implementation of coached interaction sessions

The intervention aims at changing behaviours, namely improving communication on sexuality within families. Behavioural change theories identify precursors of behavioural change. Evidence shows that health promoting interventions are more efficient when based on behavioural theories [28]. Theory of Planned Behaviour (TPB) focuses on theoretical constructs related motivational factors that determinants of the likelihood of performing a specific behaviour. This theory is an extension of the Theory of Reasonable Action (TRA) [29] which asserts that the most direct determinant of a behaviour is one’s behavioural intention that is influenced by the attitude towards performing the behaviour and the subjective norm associated with the behaviour [30]. Through recognition of the existing factors outside an individual’s control, theorists added perceived control, which explains the control beliefs concerning the presence or absence of facilitators and barriers to behavioural performance [30–32].

According to the TPB, behaviours are determined by norms, attitudes and perceived behaviour control [32, 33]. In our case, factors that inhibit open communication on sexuality within families are, amongst others, the attitude that talking about sexuality drives adolescents to sexual intercourse, the norm that adolescents should not talk about intimate things with parents; and the perceived inability to use direct language [18].

TPB focuses on individual factors influencing behaviour. However, ones behaviour highly depends on the perception of the existence of others which can or cannot approve certain conduct. Therefore we added to the concepts of TPB the assumption that interpersonal interaction is the main driver for changing norms, attitudes and perceived behaviour control [34].

### Description of the intervention

The intervention consisted of three consecutive coached sessions in which young women, aged between 15 to 24 years, and female adults, women above 25, interacted about sexuality. Identification of the participants was made through the focal point of health in the referred Municipal District. This focal point was responsible for displaying the information about the intervention among the community members, encouraging them to freely participate.

The interaction sessions took place for 3 weeks in August 2018. In total, 16 women participated in the sessions, from which eight aged 15 to 24 and eight aged between 25 and 58 years. In order to improve the exchange of sensitive experiences between women of different age, the sessions were facilitated by both a female adult and a young women. Relatives in the first degree were excluded from participating together in the sessions with the purpose of creating a safe environment to talk freely about sexual health issues. This decision does not disregard the important role that family plays regarding social norms related to sexuality as we recognize the African perspective of extended family. This means that members of the extended family or community take responsibility for young individuals [35, 36]. In Africa, sexuality is discussed with grandmother, aunt or other adults from the community, which constitutes the extended family. Among young women three were mothers, three did not complete primary school, and three were attending secondary school. From adult women, all were mothers, four of them had only attended primary school, four completed or were attending secondary school, and three were mothers of adolescents. Most of the participants earned their living through small businesses such as selling products at an informal market or near their residences. Four women were activists working with HIV positive children.

In the first session, participants interacted about puberty, starting by sharing experiences on how and what is said to children and adolescents about the transformations in this period. The second session focused on dating-related risks by exploring the perceptions about the best time to talk about dating, the role of parents, and the consequences of dating without information about the precautions to take. The third session focused on teenage pregnancy discussing amongst others about consequences, the role of the parents in preventing pregnancy, and how to cope with early pregnancy. The central point in the sessions was the reflection on communication between adults and adolescents and within families.

The sessions lasted for approximately 50 min. The sessions and interviews were held in two languages, namely Portuguese and Cichangana (local language). The sessions were facilitated by one female adult (the main researcher MF) and one young woman (a researcher assistant). The interactions were introduced by videos and photos showing young women’s experiences related to e.g. nightlife, alcohol consumption, school attendance, sexual harassment, dating including intergenerational dating, STIs and HIV prevention, pregnancy and abortion. After the introduction which lasted for about 20 min, the participants were invited to share their views in open interactive discussions about the content of the videos. Attitudes, knowledge, norms, and beliefs were shared between participants, with each participant trying to explain the real situation in their families and communities. This open interactive discussion lasted about 20 min. The last 10 min were reserved for summarizing the main aspects of the discussions. The researcher and the research assistant observed and recorded the sessions for later analysis.

### Realist Evaluation

Realist evaluation was used as a method to identify the mechanisms through which the implementation of the intervention contributed to improved communication on sexuality. The aim was to understand how, why, for whom, and under what circumstances an intervention worked. This approach allows the extrapolation of the results across different situations as it aims at formulating plausible explanations and conditions on how the intervention might work. The evaluator hypothesizes in advance the program theory, including mechanisms that are likely to operate, the contexts in which they might operate, and the expected outcomes [37–40].

The initial theory of this study intervention was as follows: **“**Young women and female adults from the same community, invited to a series of interactive sessions on sexuality issues in a safe and trustful environment, will learn from each other leading to an improved communication on sexuality within families”.

### Data collection and analysis

Data were collected through 13 in-depth interviews with participants selected from the intervention group. Of those six were young women (ages 15–24), and seven were female adults. Three refused to participate in the interviews. We are unsure about the reason for the refusal. The interviews occurred 1 week after the last session. The researchers explored participants’ experiences with the interactive sessions aiming at getting a clear understanding of how participation contributed to improved intergenerational communication on sexuality and on inhibiting and facilitating factors. The interviews guideline was initially based on the above-mentioned mechanisms from the evaluation theory and was adapted organically during the data collection process. Then, the interviews were audio-recorded and transcribed. Transcripts were imported into NVivo 12.

We conducted interviews in both Cichangana and Portuguese languages. Interviews in local language were translated into Portuguese, and then all were translated into English.

The first author structured the data following the context-mechanism-outcome (CMO) categories of the realist evaluation approach and the constructs of the Theory of Planned Behaviour [32, 33]. The codes were extensively discussed with the other authors. Critical realist grounded theory was used as a methodology for the generation of new underlying mechanisms [41].

## Results

### Characteristics of the participants

The 13 in-depth interviews with young and adult women were conducted in August 2018. Each interview lasted for approximately 1 h. The sociodemographic characteristics of the interviewee are presented in Table 1.

**Table 1:** Socio-demographic characteristics of participants

Categories	Number	Categories	Number
Age		Occupation	
*15–19*	3	student	3
*20–24*	3	informal seller	5
*25–58*	7	activist	4
		without occupation	1
Marital status		Education level	
unmarried	4	primary school	4
married	9	grade 8–10	4
		grade 11–12	5
Children			
0	3		
1–2	8		
3–6	2		

### Context, mechanisms, and outcomes

**Fig. 1:**
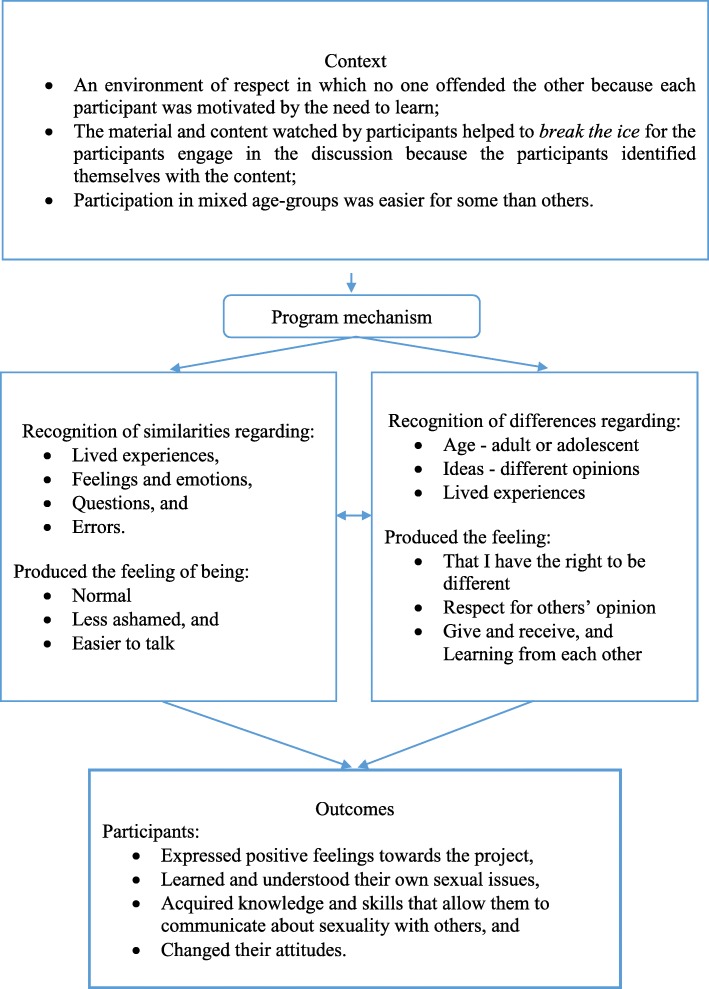
Summary of context, mechanisms, and outcomes

A schematic diagram of the context, mechanisms, and outcomes is shown in Fig. 1.

#### Outcomes

All respondents agreed on the fact that the project responded to a real need. They expressed positive feelings towards the intervention.

*I have a neighbour whose daughter got pregnant and was then kicked out from the house. If this meeting had occurred before [she had been kicked out], my neighbor would have not kicked her out.* (34 years).

*We hope to have more similar initiatives where we can learn. I liked it a lot. Every Wednesday I was waking up early, and do all my work at home to be at the sessions on time. Even my husband was asking ‘where are you going’? And I was saying: I am going to a women’s meeting*. (23 years).

*If possible, these sessions should be organized again to allow other people to participate and hear how these topics can be discussed at home. We need to grow up.* (58 years).

The interaction with others helped to understand own sexual issues. By sharing information, participants learned about contraceptive methods and STIs protection, and about languages and hidden meanings when talking about sexuality.

*I learned that there are many things [methods] easy to use [to prevent pregnancy]. If a person is not prepared to have a baby, she can use an implant or other things that can help. I also learned that for any method it is necessary to combine it with a condom*. (18 years).

*The sessions helped us. I heard my colleague saying, now I know that I should not do anything without being prepared*. (21 years).

*I learned things that my parents did not know how to teach me. For example, my mother, when I started menstruation, said that I have to avoid to be with boys. Because of that, I gave up my friendships. But now I understood that there is no need to do that. What is necessary is to avoid a romantic relationship.* (24 years).

Participants reported having acquired knowledge and skills that allow them to communicate about sexuality.

*With this experience, I can help other mothers or young women.* (56 years).

*Now I can approach my daughter and talk.* (37 years).

*I learned about communication on sexuality.* (32 years).

The sessions seem to have contributed to a change in attitudes among participants. Those participants reported on changed attitudes related to the importance of communication about sexuality, the bi-directionality of adolescent-adult communication, the ways on how men and women should be educated in sexuality and the necessity to involve men in the communication on sexuality.

*The session was like teaching us that we have to communicate with parents. They showed us the importance of dialogue between children and parents.* (16 years).

*Adults teach young people and young people also teach adults*. (25 years).

*Sometimes when I was talking with my daughter, I was doing it angrily, while it was necessary to talk lovely. Seeing me angry she was afraid to inform me about her doubts. When she got pregnant, she did not tell me the truth. So, I had to request my friend to ask her about.* (34 years).

*Mixed group of men and women to discuss [sexuality]is good. Men need to understand how dangerous they [men] are.* (16 years).

### Mechanisms

Program mechanisms ‘trigger’ or produce the outcomes of the intervention. In the initial theory, we hypothesized that the interaction sessions would foster the learning from each other which contributes to the outcome, “improved communication on sexuality”. The study allowed us to deduct two important underlying mechanisms that explain the learning from each other: the recognition of similarities and the awareness of differences.

Participants discovered that regarding sexuality they are similar to other participants of the group and to other people elsewhere.

*They [sessions of interaction] were ways of discussing things that happen. I can say, these things happen worldwide.* (24 years).

*What convinced me is the fact that I saw these things in our society.* (23 years).

This reduced the feeling of shame and eased of the resistance to talk about sexuality issues.

*I understood that there is no need to be embarrassed because all of us faced these phases: menstruation, pregnancy, abortion. The situation is different, but all of us faced these things. Each one in her specific form, but the process is the same. The difference is the way in which each body responds to the situation.* (24 years).

*First I felt embarrassed but after I realized that there were only women in the group I talked.* (18 years).

Similarities were seen at the level of experiences, feelings and emotions and participants recognized that others also lacked knowledge and skills and made similar mistakes. Women recognized i.e. similarities in the sort of information received at the first menstruation, in the attitudes regarding the first menstruation - particularly the fear of informing parents about it -, and in the relationship between mothers and daughters.

*The history of that adolescent happened with me and many adolescents now have a similar history. I took a long time without informing my mother that my menstruation cycle initiated.* (23 years).

The recognition is transgenerational. Young women and female adults discovered that they have more similarities than anticipated. It was not only young women who had little courage to inform their parents about sexuality events that were facing (previous quotation) but also adults were reluctant to talk about sexuality with young women, which can contribute to the reduction of connectedness.

*I am talking, for example, about my husband. Because when I talk about dating with my son, he says, no you must not do that because he [the son] is very young. You will motivate him to do these things.* (30 years).

Concurrently, the awareness of the differences was a second important underlying mechanism of the process. This awareness of diversity concurred strongly with values of respect and the right of being different. The respectful exchange of these differences contributed strongly to the concept of reciprocity and learning from each other.

*We talked about our experiences. As each one was telling her story, I fixed something, and I learned. From my talk, another participant also learned.* (21 years).

*Adults teach adolescents, and adolescents also teach an adult. Adults had their conclusions, and we also…They [mothers] counsel us.* (16 years).

*Mothers do not like to talk. They think about things that happened a long time ago. You know, generations are different. Mothers have to think about the current generation, where we are…they [use to] say, long time ago we did not talk with our mother because they were difficult women.* (18 years).

#### Context

Finally, this study identified contextual factors that supported or inhibited the mechanisms to produce the outcomes.

The use of visual material (videos and photos) was highly appreciated and contributed strongly to both mechanisms, recognition of similarities and the awareness of differences. The fact that some participants agreed with the content of the videos and others did not foster the debate.

*Videos and photos allowed us to talk.* (25 years).

*The way in which that girl was dancing, I did not like. It reminds me of my friend. When she dances she Twerks [sex dance moves].* (24 years).

*The mistake I found in this session is related to that girl that said she is using IUD [intra-uterine device]. I think the use of this method requires to see the age of the person.* (56 years).

The interviewees confirmed that the intervention occurred in a good and safe environment, characterized by respect for each other which is key to feeling comfortable and to expressing opinions.

*I did not feel afraid because it is not the first time to participate in a similar meeting where the discussion is about dating and pregnancy. I felt comfortable because I like to talk and discuss, to teach and learn.* (16 years).

*I liked the sessions, I felt well. Each one talked about her experience. Even those women who are young succeeded to talk. No one insulted other.* (56 years).

*In the sessions, I did not see wrong things. There, no one offended others. All of us talked. We gave our point of views. We talked about ourselves, not about others.* (24 years).

*There was big learning. I did not see any [participant] talking strangely or angry during the sessions. This shows that it was good.* (37 years).

The major obstacles for the intervention process are the taboos on sexuality added to the misconceptions on the consequences of talking about sex.

*Abortion is secret. People should not know that you induced abortion.* (24 years).

*IUD is not for adolescents. If they use this now, when they will marry and want to have a baby, these things will have blocked their fertility.* (37 years).

*It is embarrassing because talking about [sex, dating] mothers will think we are motivating adolescents.* (30 years).

*Do not talk about dating because we must not confirm for our mother that we knew these things.* (23 years).

The fact of knowing each other was differently appreciated by the participants. For some, mostly adults, the presence of known people was not considered as a barrier to talk, while others felt embarrassed. *“I knew almost all women, but this was not a barrier to talk”.* (37 years).

*I felt embarrassed because I knew some participants and I thought that they would hear my secrets.* (18 years).

The participants made recommendations regarding contextual factors that might improve the effect of the intervention, namely to create groups with mixed groups of age and gender and to have peers of the same household (mother and daughter or father and son). Apart from that, participants also expressed the wish to invite important relatives to participate in similar sessions.

## Discussion

### Moderated sessions between adults and young contribute to sexual health

This study reveals underlying mechanisms and contextual factors that explain how moderated interaction sessions between female adults and young women reduce the taboo for talking about sexuality issues.

The intervention was perceived as successful by both adults and young women. Participants at the interaction sessions confirmed that they learned from each other and that this changed the way they looked at sexuality and sexuality communication. These results are consistent with other intervention studies that demonstrated an effect of adolescent-adult interaction on cultural norms, taboos [22], behaviours and misconceptions on the link between sexuality communication and early sexual debut [23].

This outcome may be interpreted within the conceptual framework of the TPB that describes predictors for behaviour change [30–32]. The intervention might have contributed to a change in some key variables that influence the behaviour regarding intergenerational communication on sexuality. The results suggest that participants have changed their perception on the social norms on talking about sex, on their own ability to participate in such discussions and on the belief that interaction on sexuality produces positive effects on understanding own sexual issues and on an improved sexual wellbeing. According to the theory of Ajzen, these altered perspectives lead to the intention to enact differently as before.

### Mechanisms: recognition of similarities and awareness of differences

We found two, apparently contradictory, key mechanisms that produced the positive outcomes of the intervention: the recognition of similarities and awareness of differences. The effects of similarity and diversity on the performance of groups have been extensively described in scientific literature by Byrne [42]; Bennett et al. [43]; Mannix et al. [44]; Hornsey et al. [45]. On one hand, individuals are more attracted to similar others and experience more cohesion and social integration in homogeneous groups [36]. On the other hand, diversity creates an opportunity for deeper analysis and learning [44, 46]. The interaction between individuals with different backgrounds, skills, and experiences leads to an exchange of different perspectives and approaches to the problem. This intervention involved women who differ in age, sexual experiences, reproductive life (childless, mother, grandmother), marital status, schooling and occupation (e.g. HIV activists, informal sellers, students). Consequently, for a major impact of interaction sessions, it is crucial to consider thoroughly the balance between homogeneity and diversity when composing the group [44, 46].

### Facilitating and inhibiting factors: safe environment and misinformation

The main contextual factors that influenced the intervention process were related to a respectful and safe environment and to the existing taboos and myths. The respectful environment resulted from the capacity to listen to other people’s opinion and contribute with own ideas. The feeling of mutual respect within the group made each woman feel identified and satisfied with the group which facilitated the interpersonal attraction and liking [43,44,45] and motivated to contribute positively to the group process [47]. However, taboos and myths appeared to have limited young women to express certain doubts about sexuality within the group because they are not expected to use contraception or to have a boyfriend. It was also noticed that young and adult women participants have multiple erroneous ideas on sexuality issues. An interviewed participant stated that it is wrong for young women to use IUD as a contraceptive method. This is a misconception given the scientific evidence that long-action-reversible-contraceptives (LARC) are recommendable for adolescents. Fostering interaction on sexuality between people does not necessarily lead to the exchange of correct and evidence-based information. The risk that by this approach misinformation is shared within families and communities is real and cannot be avoided [17]. However, the main purpose of the intervention was not spreading correct information but sharing ideas, perspectives, opinions, and beliefs starting from the assumption that interpersonal interaction is a driving mechanism towards behavioural change. The interaction itself prevails over the content. The basic principle is that all what people say is meaningful deserves respect and is worth to be communicated. Disclosure of those meanings is enriching and contributing to a larger understanding. Basically, we believe that at the long term the agreed truth will rule in concordance with the ideas of the philosopher Habermas who relates the meaning of truth with the outcome of a universal, rational consensus (consensus theory) [48]. To disclose this truth, more communicative actions are needed. It is unlikely that misconceptions, myths, and taboos will disappear after a few sessions. These interventions should rather be considered as a trigger point for strategies that impulse intergenerational communication within communities and families.

### Innovative intervention

From the early 90’s the Mozambican government implemented several interventions with the aim to improve the sexual health of young people. A variety of methods were used: face to face education, youth-friendly health services, peer-led education, digital communication (SMS, facebook, voice calls, interactive platforms, and virtual counselling) [24, 49, 50]. As far as we know this piloted intervention appears to be a unique experience in Mozambique in using facilitated interaction between young people and adults from the community as a tool to promote intergenerational communication on sexuality.

### Limitations and strengths

Considering its nature, this realist case study does not claim to produce universally applicable findings. Cultural norms, which are strongly embedded in the Mozambican context, may have influenced the participants to give socially desirable responses.

The majority of women aged 15 and above living at this community were illiterate. However, most of the participants of this pilot were at or completed secondary school. It is a limitation of this intervention study that there were almost no illiterate women included, what leads to a selection bias.

This pilot intervention was implemented at a community level joining non-familiar participants. Inducing communication about sexuality at the community might contribute to sexuality communication within families living in the same context and sharing similar attitudes, beliefs and norms. By involving non-familiar participants, we contributed to expanding the experience to different community unities (families).

Boys and men were not included in the intervention. This is a limitation as women’s sexual health is largely influenced by men’s behaviour e.g. the ability to use contraceptives and gendered norms. We opted for exclusive female meetings intending to create an environment where women feel free to talk about sensitive topics.

The sample size was limited. However, we did manage to include women with varying backgrounds.

Notwithstanding these limitations, the study has shown mechanisms and conditions that might be suitable for similar projects aiming at promoting intergenerational communication within communities and families in Mozambique and elsewhere. The inclusion of boys and the acceptance of persons from the same household are the most important recommendation for similar interventions in the future that aim to improve communication on sexuality.

## Conclusions

Our research explored how participation at three coached sessions in which female adults and young women interacted about sexuality, contributes to an improved trans-generational communication on sexuality. Underlying mechanisms were related to the recognition of similarities and the awareness of differences. The safe and respectful environment of the interaction was mentioned as the main supporting factor. The existing cultural taboos and misconceptions on sexuality limited the intervention process. Taken together, we believe that this pilot intervention can contribute to future strategies on promoting sexual and reproductive health.

## Data Availability

Dataset is part of a broader research for the fulfillment of the requirements for the degree of Doctor of Philosophy of the first author. It may be shared, if necessary, on reasonable request.

## Abbreviations

HIV: Human immunodeficiency virus
STIs: Sexually transmitted infections
